# Interleukin 6 receptor is not directly involved in regulation of body weight in diet-induced obesity with and without physical exercise

**DOI:** 10.3389/fendo.2022.1028808

**Published:** 2022-10-27

**Authors:** Anna Rita Minafra, Alexandra Chadt, Puyan Rafii, Hadi Al-Hasani, Kristina Behnke, Jürgen Scheller

**Affiliations:** ^1^ Institute of Biochemistry and Molecular Biology II, Medical Faculty, Heinrich-Heine-University, Düsseldorf, Germany; ^2^ Institute for Clinical Biochemistry and Pathobiochemistry, German Diabetes Center, Medical Faculty, Heinrich-Heine-University, Düsseldorf, Germany; ^3^ German Center for Diabetes Research Deutsches Zentrum für Diabetesforschung e.V. (DZD), Partner Düsseldorf, München, Neuherberg, Germany

**Keywords:** interleukin 6 (IL-6), obesity, exercise, diet, mouse model

## Abstract

High level of interleukin 6 (IL-6), released by adipocytes in an obesity-induced, low grade inflammation state, is a regulator of insulin resistance and glucose tolerance. IL-6 has also regenerative, anti-inflammatory and anti-diabetogenic functions, when secreted as myokine by skeletal muscles during physical exercise. IL-6 mainly activates cells *via* two different receptor constellations: classic and trans-signalling, in which IL-6 initially binds to membrane-bound receptor (IL-6R) or soluble IL-6 receptor (sIL-6R) before activating signal transducing gp130 receptor. Previously, we generated transgenic soluble IL-6 receptor ^+/+^ (sIL-6R^+/+^) mice with a strategy that mimics ADAM10/17 hyperactivation, reflecting a situation in which only IL-6 trans-signalling is active, whereas classic signalling is completely abrogated. In this study, we metabolically phenotyped IL-6R deficient mice (IL-6R-KO), sIL-6R^+/+^ mice and wild-type littermates fed either a standard chow (SD) or a high-fat diet (HFD) in combination with a 6-weeks treadmill exercise protocol. All mice were subjected to analyses of body weight and body composition, determination of blood glucose and insulin level under fasting conditions, as well as determination of substrate preference by indirect calorimetry. Neither classic IL-6 nor trans-signalling do influence the outcome of diet-induced obesity, insulin sensitivity and glycaemic control. Furthermore, IL-6R deficiency is not impairing the beneficial effect of physical exercise. We conclude that the IL-6R does not play a requisite role in regulation of body weight and glucose metabolism in diet-induced obese mice.

## Introduction

In the last two decades, obesity has been described not only as elevated amount of fat cells caused by excess of nutrients and a low degree of physical inactivity, but also associated with an inflammatory state. The transition from healthy lean to obese adipose tissue is accompanied by a chronic low-grade inflammation and immune dysregulation, as well as the enhanced release of pro-inflammatory cytokines, which can consequently interfere with peripheral insulin signalling and glucose metabolism. Among others, IL-6 has been frequently associated with the impaired immune control in obese adipose tissue (1).

Among several studies, Wallenius et al. showed that mice lacking IL-6 developed mature-onset obesity, associated with a disturbed carbohydrate and lipid metabolism (2). These data were subsequently supported by Matthews et al., who observed increased body weight, impaired glucose tolerance and exacerbated insulin resistance in IL-6 KO mice (3). Moreover, enhanced inflammation in liver and skeletal muscles and insulin resistance was observed in hepatocyte-specific IL-6R deficient animals (4), as well as increased insulin resistance in whole-body IL-6 KO mice (5) and increased body weight in astrocyte-specific IL-6 deficient mice (6). In contrast, adipocyte-specific deletion of IL-6 in the context of diet-induced and genetic obesity had no effect on body weight and fat content, glucose tolerance and insulin resistance (7) or, in another study, it determined slightly reduced high fat diet (HFD)-induced glucose intolerance (8).

Taken together, it is still unclear whether IL-6 is a primary trigger for the development of obesity and insulin resistance or whether it is actually required to counteract the increased inflammation associated with obesity. Additionally, this intricate scenario is complicated further by the observed beneficial effect of IL-6 produced by skeletal muscles following physical exercise.

Indeed, during intense exercise, both IL-6 mRNA and protein levels increase in skeletal muscles (9, 10) and plasma IL-6 rises up to 100-fold (11). In addition, there are many lines of evidence that IL-6 has also regenerative effects, transiently downregulates immune function and can actually protect from obesity and insulin resistance. Indeed, during physical exercise, IL-6 promotes blood glucose disposal and blood glucose uptake in skeletal muscles by stimulating cell surface glucose transporter 4 (GLUT4) translocation in muscle cells (1). In addition, it may increase fatty acid uptake, lipolysis and free fatty acids release from adipocytes and skeletal muscles, respectively (12, 13). Moreover, IL-6 has been shown to stimulate pancreatic insulin production and insulin sensitivity in peripheral tissues, including skeletal muscle and adipose tissue, together with enhancing skeletal muscle hypertrophy and bone remodelling (14).

Mechanistically, two main signalling pathways can be activated by IL-6. In the classic signalling pathway, IL-6 binds to its membrane-bound receptor (IL-6R), followed by dimerization of glycoprotein 130 (gp130) and activation of JAK/STAT, MAPK, and PI3K/AKT (15). In the *trans*-signalling pathway, IL-6 can bind soluble IL-6 receptor (sIL-6R) molecules which are generated *via* ectodomain shedding by metalloproteases (ADAM-10 and ADAM-17) (16) or through alternative splicing of IL-6R mRNA (17).

Of note, activation of classic IL-6 signalling is limited to specific tissues, since IL-6R is only expressed in distinct cell types, such as immune cells and hepatocytes. Some studies suggest that IL-6R might be expressed also in adipocytes and myocytes (18–20). Thus, it is not yet clear whether metabolic functions of IL-6 mainly rely on classic or trans-signalling. Accordingly, here, we metabolically characterized the previously generated IL-6 trans-signalling mice, which were genetically engineered to execute IL-6 trans-signalling, with a strategy that mimics ADAM10/17 hyperactivation generated by Cre-mediated deletion of the genetic information coding for the transmembrane and intracellular domain of the IL-6R. Due to this lack, IL-6R is directly secreted as soluble IL-6R. Consequently, these mice selectively execute trans-signalling, whereas classic signalling is abrogated (21).

Here, we analyzed IL-6R deficient mice in diet-induced obesity and physical exercise. Albeit also IL-6 deficient mice have shown contradictory results, IL-6R deficient mice phenotypically strongly deviate from IL-6 deficient mice in wound healing (22) but not in liver regeneration (21). We were, therefore, interested if IL-6R deficient mice phenocopy IL-6 deficient mice in diet-induced obesity. Furthermore, we hypothesize that IL-6 trans-signalling is mainly involved in glucose metabolism regulation in diet-induced obesity and may be affected by physical activity, while classic signalling is linked to a homeostatic regulation.

## Results

### Specific activation of IL-6 trans-signalling and deficiency of classic and trans-signalling does not influence body composition of mice under standard diet

**Figure 1:**
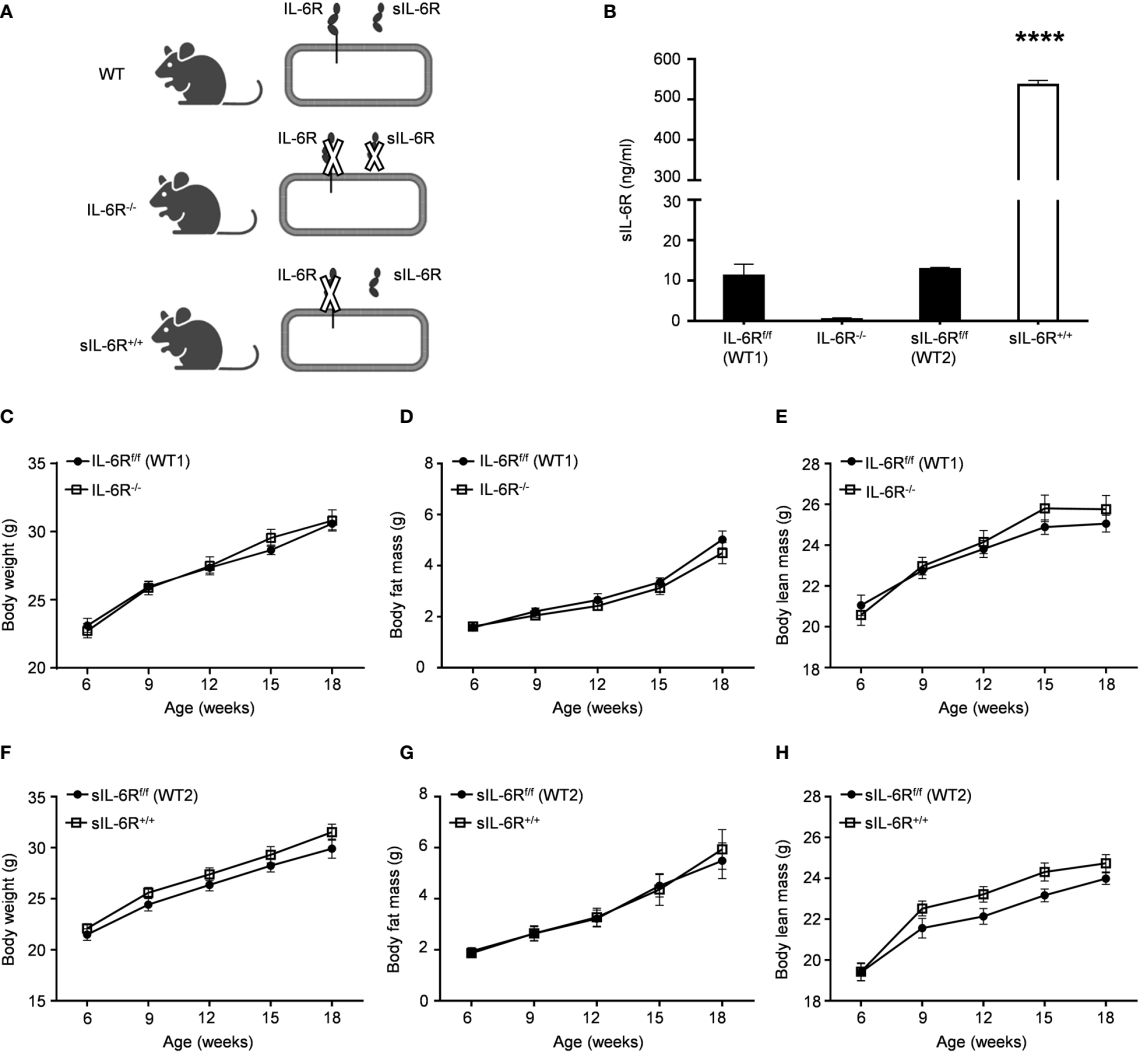
IL-6R signalling does not regulate body weight and body composition in normal condition. **(A)** Schematic representation of IL-6 signalling activation in WT, IL-6R^-/-^ and sIL-6Rf/f mouse models. **(B)** Circulating level of IL-6 receptor was measured by ELISA in littermates IL-6R^f/f^ (WT) and IL-6R^-/-^ and in littermates sIL-6R^f/f^ (WT) and sIL-6R^+/+^ (n=5-6). **(C)** Body weight, **(D)** body fat and **(E)** body lean in littermates IL-6R^f/f^ (WT) (n=11-13) and IL-6R^-/-^ (n=14-18). **(F)** Body weight, **(G)** body fat and **(H)** body lean in littermates sIL-6R^f/f^ (WT) (n=9-11) and sIL-6R^+/+^ (n=8-11) at the indicated weeks of age, maintained on standard diet (SD) and in resting condition. Data are presented as means ± SEM; ****p < 0.0001.

We first generated transgenic *soluble* IL-6R^+/+^ (sIL-6R^+/+^) mice, as previously described (21). Shortly, in these mice, the endogenous hyper-activation of ADAM10/17 is mimicked by Cre-mediated deletion of the genetic information coding for the transmembrane and intracellular domain of the IL-6R, reflecting a situation in which only trans-signalling is active, whereas classic signalling is abrogated. According to this mouse model, membrane-bound IL-6R is entirely converted into soluble IL-6R (sIL-6R) allowing only endogenous IL-6 *trans*-signalling. To endorse our model, we initially tested the plasma level of sIL-6R, using wild-type (WT) littermate controls and a mouse model with complete deficiency of IL-6R (IL-6R-KO), where both IL-6 trans and classic signalling are abrogated (**Figure 1A**) . As expected, the circulating level of sIL-6R was increased in sIL-6R^+/+^ mice (approximately 536 ng/ml) and completely absent in IL-6R deficient mice in comparison to the strain control mice with 12.8 ng/ml for sIL-6R^f/f^ (wild-type 1 for sIL-6R^+/+^) and 11.2 ng/ml for IL-6R^f/f^ (wild-type 2 for IL-6R^-/-^)) (**Figure 1B**).

**Figure 2:**
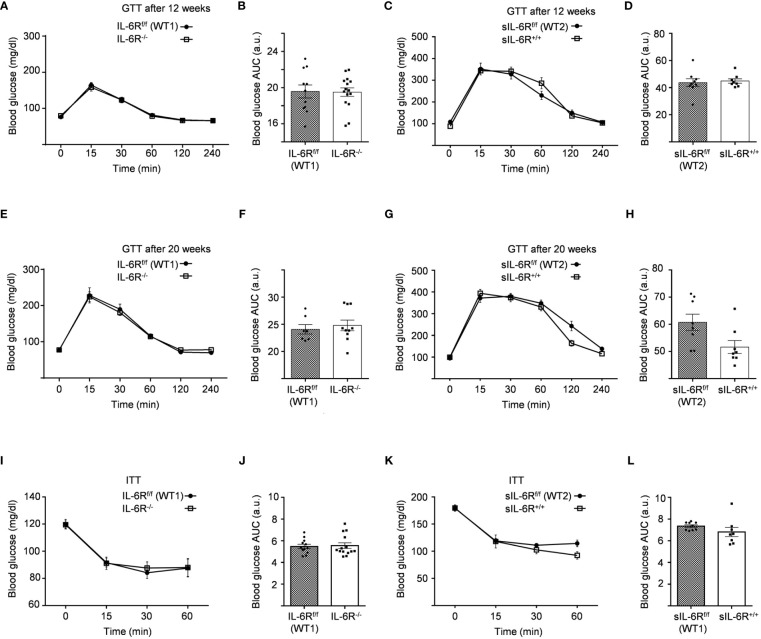
Intraperitoneal glucose tolerance (GTT) and insulin tolerance (ITT) tests are not impaired in IL-6R deficient mice. **(A)** GTT: at 12 weeks of age, glucose level was measured at indicated time points (min) after 16 hours fasting and post intraperitoneal glucose injection in littermates IL-6R^f/f^ (WT) (n=11) and IL-6R^-/-^ (n=15). **(B)** Total area under the curve (AUC). **(C)** GTT: glucose level was measured at indicated time points (min) after 16 hours fasting and post intraperitoneal glucose injection in littermates sIL-6R^f/f^ (WT) (n=9) and sIL-6R^+/+^ (n=8). **(D)** Total area under the curve (AUC). **(E-G)** Intraperitoneal glucose tolerance test (GTT) at 20 weeks of age (n=7-10). **(F-H)** Total area under the curve (AUC). **(I)** ITT: at 14 weeks of age, glucose level was measured at indicated time points (min) post intraperitoneal insulin injection in littermates IL-6R^f/f^ (WT) (n=12) and IL-6R^-/-^ (n=14). **(J)** Total area under the curve (AUC). **(K)** ITT: at 14 weeks of age, glucose level was measured at indicated time points (min) post intraperitoneal insulin injection in littermates sIL-6R^f/f^ (WT) (n=9) and sIL-6R^+/+^ (n=8). **(L)** Total area under the curve (AUC). All mice were fed with standard CHO diet and were in resting condition. Data are presented as mean ± SEM.

We monitored body composition in resting condition and under standard diet (SD) in IL-6 trans-signalling mice, IL-6R deficient mice and the appropriate wild-type littermates. After 18 weeks, we did not observe any differences in body weight, body fat and lean mass due to different genotypes but a similar progressive tendency throughout the 12 weeks (**Figures 1C-H**). Next, we tested the effect of sIL-6R over-expression and IL-6R deficiency on glucose and insulin tolerance under standard diet by intraperitoneal glucose tolerance test after 16 h fasting at 12 weeks and at older age of 20 weeks. There was no detectable variation in glucose disposal related to different IL-6 signalling activation (**Figures 2A-H**). We also measured blood glucose levels following an insulin injection for 15, 30, and 60 minutes, with no differences observed for the different genotypes in insulin sensitivity (**Figures 2I-L**). In summary, we did not observe any influence of specific activation of IL-6 trans-signalling and deficiency of IL-6R classic and trans-signalling on body weight gain, glucose and insulin tolerance following standard diet.

### IL-6R signalling does not regulate basal metabolism in resting conditions

**Figure 3:**
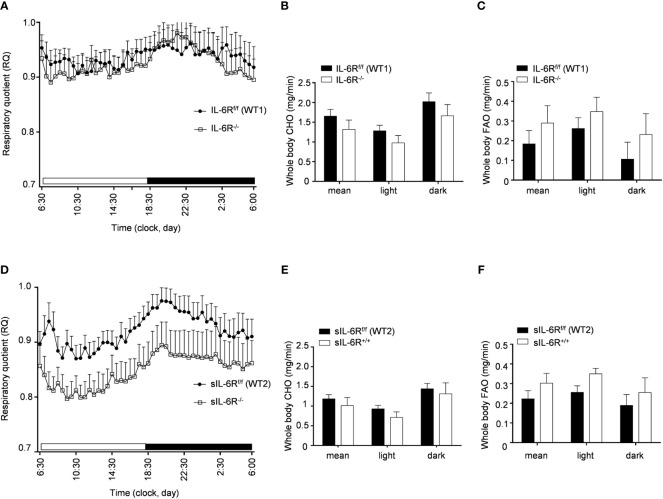
Indirect calorimetry parameters are not altered in IL-6R deficient and sIL-6R mice in resting conditions. **(A)** After 24h of adaption period, respiratory quotient (RQ) was measured as quotient of VCO_2_ and VO_2_ over 12h light (06:00-18:00) and dark (18:00-06:00) phases in littermates IL-6R^f/f^ (WT) (n=11) and IL-6R^-/-^ (n=11) and **(D)** in littermates sIL-6R^f/f^ (WT) (n=11) and sIL-6R^+/+^ (n=9), fed with standard diet and in resting condition. Glucose: RQ=1; Fat: RQ=0.7: Protein: RQ=0.82. **(B-E)** Whole body carbohydrate oxidation rate (calculated as $4.585\times \dot{V}CO_{2}(l/min)-3.226\times \dot{V}O_{2}(l/min)$ ) as average of 24h and during light and dark phases. **(C-F)** Whole body fat oxidation rate (calculated as $1.695\times \dot{V}O_{2}(l/min)-1.701\times \alpha \dot{V}CO_{2}(l/min)$ ) as average of 24h and during light and dark phases. Data are presented as mean ± SEM.

In human and animal trials, indirect calorimetry is frequently used to assess the total energy expenditure together with the respiratory exchange ratio (RER), calculated as ratio between O_2_ and CO_2_ consumption, index of glucose, protein or fat oxidation as a fuel source (23). We therefore tested the basal metabolic capacity in sIL-6R^+/+^ and IL-6R deficient mice. As expected, we noticed a slight increase of RER during the dark phase, index of a metabolic shift towards glucose oxidation (**Figures 3A-D**). Despite a decreased respiratory quotient in sIL-6R^+/+^ in comparison to WT mice in both light and dark phases, no correlation with different genotypes was statistically significant. Whole carbohydrate oxidation rates were partially elevated while fatty acids oxidation rates were moderately reduced in appropriate WT controls compared to IL-6R deficient and sIL-6R^+/+^ mice, but the differences were not statistically significant (**Figures 3B, C, E, F**). Whole carbohydrates and fatty acids oxidation rate were calculated as previously described (24). From these data we conclude that nor IL-6 classic or trans-signalling is influencing the basal metabolism under resting conditions.

### Diet-induced obesity and treadmill exercise does not induce changes in body composition in sIL-6R^+/+^ and IL-6R deficient mice

**Figure 4:**
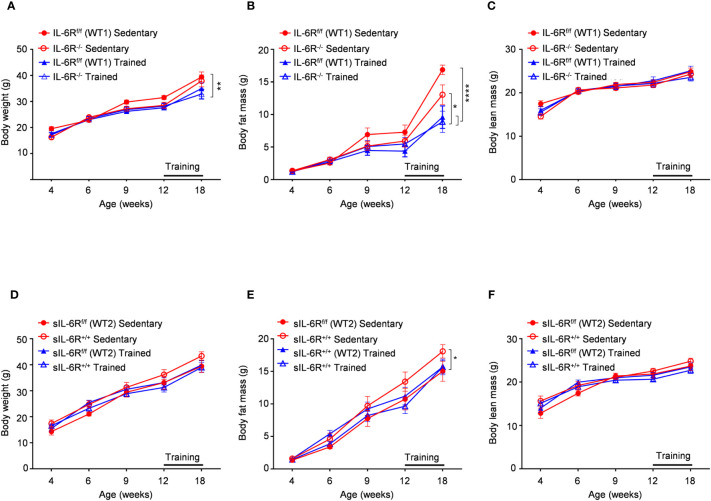
Body weight and body composition are not influenced by IL-6R signalling during high fat diet and physical exercise. All mice were fed with high fat diet from 4 weeks of age. 12- to 18-week-old mice exercised five days per week on a treadmill, progressively increasing the intensity and duration of exercise. **(A-D)** Body weight, **(B-E)** body fat and **(C-F)** body lean in littermates IL-6R^f/f^ (WT) (n=6-10) and IL-6R^-/-^ mice (n=8-9) and in littermates sIL-6R^f/f^ (WT) (n=8) and sIL-6R^+/+^ (n=8), divided in sedentary (red) and trained group (blue), at indicated time points (weeks). Data are presented as means ± SEM; *p < 0.05, **p < 0.01, ****p < 0.0001.

It has been shown that IL-6 has an important role in obesity and insulin resistance but also is cardinal in driving the beneficial effects of physical exercise in glucose and insulin sensitivity as well as body composition (1). Therefore, sIL-6R trans-signalling mice, IL-6R deficient mice and appropriate wild-type mice were fed with high fat diet (HFD) starting from week 4 until week 18 including a treadmill protocol after 9 weeks of high fat diet for the total duration of 6 weeks, 5 days per week. HFD feeding determined an increase in body weight and changes in body composition trajectory in comparison to SD. A similar trend was observed in body weight and body composition before starting the treadmill training. After 6 weeks of training, a significant increase of body weight was observed in appropriate WT mice compared to trained WT and IL-6R deficient mice, due to beneficial effect of exercise, but no differences were attributable to different genotype (**Figure 4A**). After training, we observed a reduction in body fat mass but not in lean mass (**Figures 4B, C**). We did not observe any significant change in body weight, body fat and lean mass in sIL-6R^+/+^ and control mice after training (**Figures 4D-F**). Taken together, no differences in energy metabolism due to IL-6R genotype were underlined following high-fat diet and physical exercise.

### IL-6 signalling does not rescue HFD-induced hyperglycaemia before and after training

**Figure 5:**
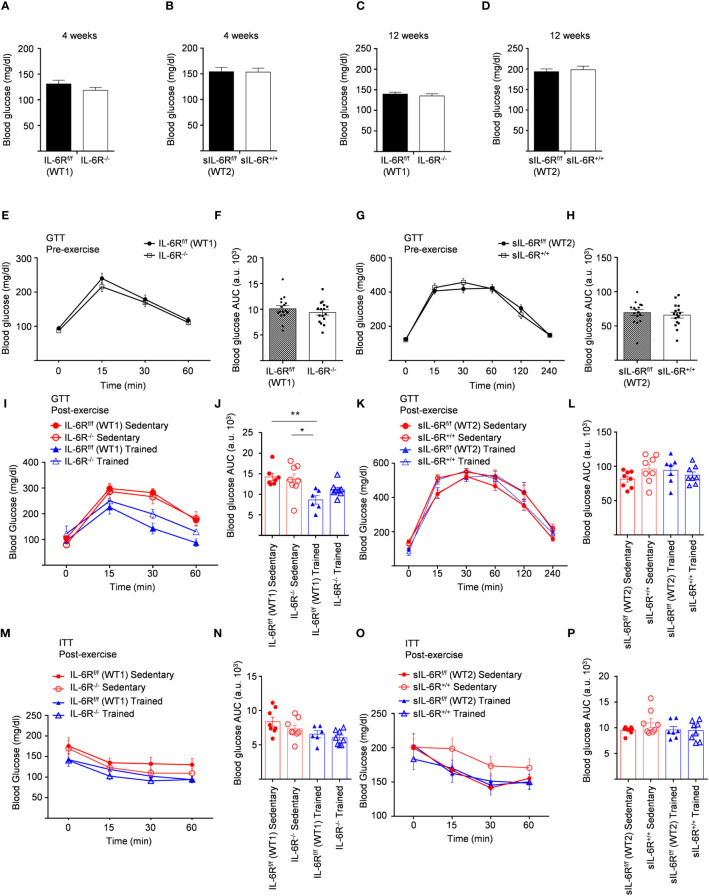
Unimpaired glucose and insulin tolerance in sIL-6R^+/+^ and IL-6R-KO mice following high fat diet and treadmill exercise. All mice were fed with high fat diet from 4 weeks of age. 12- to 18-week-old mice exercised five days per week on a treadmill, progressively increasing the intensity and duration of exercise. **(A)** FBG: fast blood glucose level was measured at 4 weeks of age in littermates IL-6R^f/f^ (WT) (n=11) and IL-6R^-/-^ (n=16) and **(B)** in littermates sIL-6R^f/f^ (WT) (n=16) and sIL-6R^+/+^ (n=16). **(C, D)** FBG at 12 weeks of age, after 8 weeks of high-fat diet (HFD) (n=15-16). **(E-I)** GTT: blood glucose level was measured pre and post training at indicated time points (min) after 16 hours fasting and post intraperitoneal glucose injection in littermates IL-6R^f/f^ (WT) and IL-6R^-/-^ (pre-training: n=16 WT and n=16 KO; post-training: n=7 WT and n=8 KO sedentary (red) and n=6 WT and n=8 KO trained (blue)). **(F-L)** Total area under the curve (AUC). **(G-K)** GTT in littermates sIL-6R^f/f^ (WT) and sIL-6R^+/+^ (pre-training: n=16 WT and n=16 sIL-6R^+/+^; post-training: n=8 WT and n=8 sIL-6R^+/+^ sedentary (red) and n=8 WT and n=8 sIL-6R^+/+^ trained (blue)). **(H-L)** Total area under the curve (AUC). **(M-O)** ITT: at 15 weeks of age, blood glucose level was measured at indicated time points (min) post intraperitoneal insulin injection in littermates IL-6R^f/f^ (WT) and IL-6R^-/-^ (sedentary (red): n=8 WT and n=8 sIL-6R^+/+^; trained (blue): n=6 WT and n=8 sIL-6R^+/+^) and in littermates sIL-6R^f/f^ (WT) and sIL-6R^+/+^ (sedentary (red): n=8 WT and n=8 sIL-6R^+/+^; trained (blue): n=7 WT and n=8 sIL-6R^+/+^). **(N-P)** Total area under the curve (AUC). Data are presented as mean ± SEM. *p < 0.05, **p < 0.01.

IL-6 signalling has been reported to be involved not only in regulation of weight loss but also in improved glucose and insulin sensitivity induced by physical activity (14). Considering that, fasting blood glucose was measured at the beginning of the HFD at 4 weeks and no variations between genotypes were observed (**Figures 5A, B**). We repeated the same experiments at the beginning of the training at 12 weeks and similarly glucose level was not impaired (**Figures 5C, D**). HFD led to an increased blood glucose level after intraperitoneal glucose tolerance test measured at 11-week-old before training compared to SD-fed mice but no significant differences were observed between the genotypes (**Figures 5E-H**).

To test whether exercise training has any impact, equally, glucose in addition to insulin tolerance was determined in high-fat diet-fed and trained sIL-6R^+/+^ and IL-6 deficient mice and littermate controls. Physical exercise significantly improved glucose tolerance in IL-6R littermate WT mice but only slightly in IL-6 deficient mice (**Figures 5I, J**), while no differences were observed between sIL-6R^+/+^ and littermate control mice (**Figures 5K, L**). Likewise, insulin tolerance after treadmill training in IL-6R deficient and sIL-6R^+/+^ mice was comparable to the sedentary group (**Figures 5M–P**).

In brief, lack of membrane-bound and soluble IL-6R did not regulate fast blood glucose level following 8 weeks of high-fat diet and did not influence glucose and insulin tolerance tests during high-fat diet and after physical exercise.

### Il-6 signalling does not influence basal metabolism in HFD-fed mice before and after training

To better understand the consequences of high-fat diet as well as the impact of physical exercise on the whole-body metabolism in sIL-6R^+/+^ and IL-6 deficient mice, we assessed substrate utilization and whole-body carbohydrate and fatty acid oxidation rate two weeks before the start of the treadmill training and after 4 weeks of exercise.

**Figure 6:**
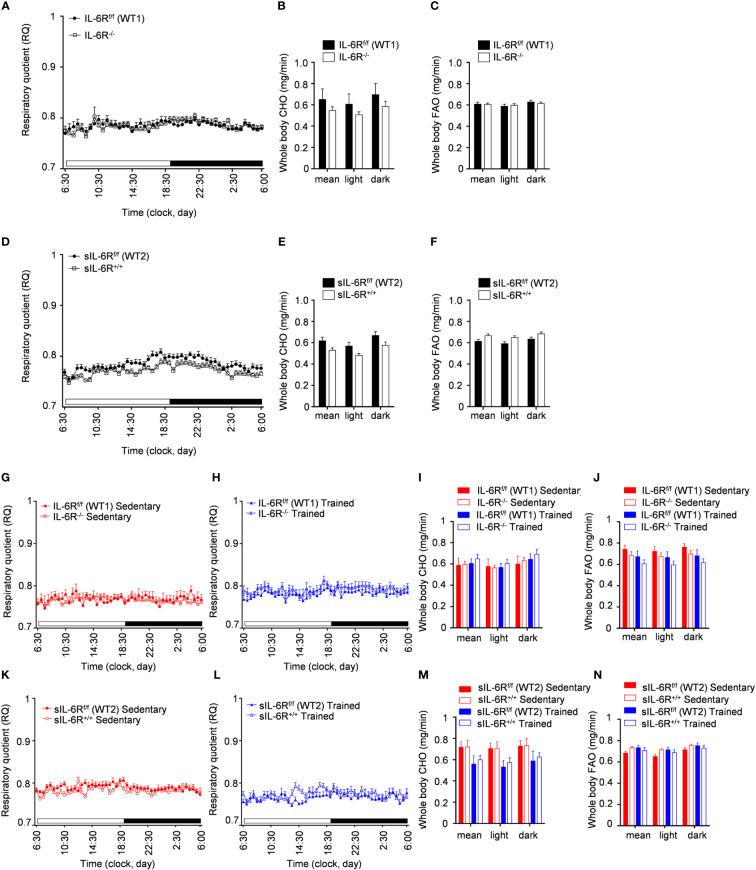
Indirect calorimetry measurements during HFD and post treadmill exercise. **(A)** After 24h of adaption period, respiratory quotient (RQ) was measured as quotient of VCO_2_ and VO_2_ over 12h light (06:00-18:00) and dark (18:00-06:00) phases in littermates IL-6R^f/f^ (WT) (n=17) and IL-6R^-/-^ (n=17) and **(D)** in littermates sIL-6R^f/f^ (WT) (n=16) and sIL-6R^+/+^ (n=17), fed with high-fat diet and in resting condition, at 10 weeks of age. Glucose: RQ=1; Fat: RQ=0.7: Protein: RQ=0.82. **(B-E)** Pre-training whole body carbohydrate oxidation rate (calculated as $4.585\times \dot{V}CO_{2}(l/min)-3.226\times \dot{V}O_{2}(l/min)$ ) as average of 24h and during light and dark phases. **(C-F)** Pre-training whole body fat oxidation rate (calculated as $1.695\times \dot{V}O_{2}(l/min)-1.701\times \dot{V}CO_{2}(l/min)$ ) as average of 24h and during light and dark phases. **(G-K)** Post-training RQ, at 16 weeks of age, in sedentary groups (red) (n=8-10) and **(H-L)** in trained group (blue) (n=6-8). **(I-M)** Post-training whole body carbohydrate oxidation rate. **(J-N)** Post-training whole body fat oxidation rate. Data are presented as mean ± SEM.

We found that substrate utilization was not influenced by physical exercise and was not different between sIL-6R^+/+^ and IL-6R deficient mice and respective WT mice (**Figures 6A, D, G, H, K, L**). Based on our results shown in **Figures 6B, C, E, F, I, J, M, N**, IL-6R signalling did not regulate the whole-body carbohydrate and fatty acid oxidation and they are not significantly influenced by exercise.

In conclusion, the respiratory quotient and the carbohydrate and fatty acid basal metabolism are not changed in high-fat diet fed IL-6R deficient mice and following treadmill training.

## Discussion

Using IL-6R deficient and sIL-6R^+/+^ trans-signalling mice, we analysed the specific role of IL-6 classic and trans-signalling in diet-induced obesity and physical exercise. In contrast to previous studies, mainly centred around IL-6 KO mouse models, our data suggested that both classic and trans-signalling have no considerable impact on body weight increase and distorted glucose metabolism following standard CHO diet and/or high-fat diet. Interestingly, sIL-6R^+/+^ mice showed modest increase in body weight in comparison to controls under CHO diet, majorly attributable to enhanced body lean mass; this phenomenon could be partially due to the observed gain of fatty acid utilization during light and dark periods that could explain the lack of increase in adipose tissue. Moreover, we did not observe any impaired blood glucose level after 9 weeks of HFD in IL-6R deficient mice and respective wild-type control. Lastly, deficiency of IL-6R did not critically regulate the beneficial effects of physical exercise on body weight, body composition, glucose and insulin tolerance, as well as basal metabolism.

For the first time in the context of obesity, we characterized the sIL-6R^+/+^ mouse model (21), an approach to simulate ADAM10/17 hyperactivation for the specific target protein IL-6R by a Cre-mediated deletion of the genetic sequence coding for transmembrane and intracellular domains that consequently lead to specific trans-signalling execution. This is a unique system to study solely the IL-6 trans-signalling, without the classic signalling activation, during patho-physiological conditions, including, in our case, obesity associated with insulin signalling and glucose metabolism distortion, as well as physical exercise. Based on our data, following high-fat diet, circulating sIL-6R is increased up to 48-fold in sIL-6R^+/+^ mice compared to appropriate littermate controls, suggesting that our model specifically executes trans-signalling and abrogates classic signalling. To support our statement, we have shown that mRNA levels of IL6-R in sIL-6R^fl/fl^ and IL-6R^fl/fl^ were comparable to wild-type mice while a significant increase was detected in sIL-6R^+/+^, and immunochemistry data additionally highlighted IL-6R being detectable in wild-type but not in IL-6R deficient and sIL-6R^+/+^ mice, demonstrating how membrane-bound IL-6R is being converted to sIL-6R and rapidly secreted (21). Collectively, this explains the notable increase of sIL-6R levels in our mouse model.

The present study was designed to clarify whether IL-6R deficient mice phenocopy the IL-6 deficient mice in diet-induced obesity and physical exercise exposure. The majority of studies linked IL-6 deficiency to development of obesity, glucose intolerance and insulin resistance, such as Wallenius et al., who indicated IL-6 KO mice to developed mature-onset obesity and insulin resistance (2), although a few years later Di Gregorio et al. reported no differences in obesity in 8 months old mice and after 3 months of high-fat diet (25).

Remarkably, our data did not show a similar phenotype between IL-6 KO and IL-6 receptor KO mice, since we did not observe any increase in body weight, changes in body composition or alterations of glucose and insulin sensibility or variations in basal metabolism, e.g. indirect calorimetry measurements. A similar scenario has been observed by McFarland-Mancini et al., where mice lacking IL-6R showed different phenotype compared to IL-6-deficient mice in delayed wound healing process, despite some similarities in inflammatory deficits (22). Of note, in this study, the combined deficiency of IL-6 and IL-6R had the similar phenotype than IL-6R. Although we did not provide comparative data for IL-6 KO, due to the high number of studies already reported in literature, we speculate that IL-6R KO plays a minor role in development of the phenotypes compared to IL-6 KO. On this basis, a plausible explanation of our adverse results could be a previously unidentified function of IL-6R that does not involve IL-6 but other cytokines or receptors. This would highlight the feasibility of the hypothesis previously formulated by McFarland-Mancini et al. that IL-6 might execute its function binding to a different receptor. Furthermore, one cannot exclude a compensatory mechanism that involves other cytokines, belonging to the IL-6 family. For instance, Schuster et at. revealed that human CNTF as well can bind and activate signalling *via* IL-6R, although with an affinity 50-fold inferior to IL-6 (26).

Another important factor to be considered is the genetic background differences that could affect the results. There are many examples of how genetic background could cause opposite effects on metabolism, for instances different murine strains have opposing effects on muscle and liver insulin sensitivity (27). It cannot be excluded that there are additional genetic variations into the mouse lines, diverse breeding strategies, divergent use of control animals (littermates or general WT), different age, as well as different methodology, environmental or dietary factors that could play a key role in causing variances between the studies. Notably, we observed surprising differences between the two control strains IL-6R^f/f^ and sIL-6R^f/f^ with respect to body weight and body composition, as well as blood glucose levels and RQ, equally in standard condition and after high-fat diet and physical exercise, independent of IL-6R genotype, with the same C57/BL6 background and identical experimental techniques. This underlines the importance of comparing the results with littermate controls rather than universal WT mice.

Together with genotype and environmental factors, an additional variable that could influence the different pathophysiology of obesity in different mice lines is the gut microbiota. It has been shown that diet-induced obese mice have different amounts of the two dominant bacterial divisions, the Bacteroidetes and the Firmicutes, and this affects the metabolic profile, leading to increased capacity for dietary energy harvest and higher body fat contents (28).

In summary, nor membrane-bound IL-6R and/or sIL-6R seems to be involved in regulating glucose and insulin metabolism in diet-induced obesity, although previous reports defined IL-6 as the major trigger of inflammation in adipose tissue in obese conditions and consequent glucose and insulin intolerance in peripheral tissues. Our results add new crucial information to what is the already known as the complex scenario related to the role of IL-6 signalling in the above mentioned mechanisms. This paves the way to address new questions that need further investigations regarding a plausible novel mechanism of IL-6R in adipocytes and skeletal muscles.

## Material and methods

### Animals

All experiments were approved by the Ethics Committee of the State Office for Nature, Environment and Consumer Protection NRW (LANUV, North Rhine-Westphalia, Germany – 84-02.04.2020.A278) and conducted at the animal facility of the German Diabetes Center. Unless otherwise specified, three to six mice per cage were housed at 22°C with a 12-hour light–dark cycle and free access to food and water. Following weaning, male animals were fed a regular chow diet (Ssniff, Soest, Germany, 11% Fat) or fed with high fat diet (HFD, Research diet, rodent, 60 kcal% Fat) from the age of 4 weeks and used for experiments from the age of 4 to 21 weeks.

Floxed sIL-6R and global sIL-6R^+/+^ mice were generated as previously described (21). IL-6R^-/-^ mice (22) were obtained from the Heinrich-Heine University of Düsseldorf’s animal facility ZETT – Zentrale Einrichtung für Tierforschung und wiss. Tierschutzaufgaben.

### Analysis of body weight and body composition

Body weight was measured every 2-3 weeks with an electronic scale (Sartorius), and body composition (body fat and lean mass) was determined by a nuclear magnetic resonance spectrometer (Bruker-Minispec NMR-Analyzer mq10; Bruker Optics).

### Fast blood glucose tolerance test

Mice fasted for 6 h before the experiments. Fast blood glucose level was measured from the tail tip. This experiment has been performed at the age of 4 weeks and 12 weeks.

### Intraperitoneal glucose tolerance test

Mice were fasted for 16 h before the experiment and subsequently were injected intraperitoneally with sterile glucose (2 g/kg body weight, 20% solution, 10 µL/g). Basal blood glucose was determined at the tail tip at 0, 15, 30, 60, 120 and 240 minutes after injection. This experiment has been performed at the age of 12 and 20 weeks, under CHO-diet, and at the age of 11 weeks (pre-exercise) and 17 weeks (post-exercise), under high fat diet.

### Intraperitoneal insulin tolerance test

Subsequently to blood glucose level measurement from the tail tip, mice were injected intraperitoneally with 10 µL/g insulin (Actrapid, Novo Nordisk, 100 U/ml). Basal blood glucose was determined from the tail tip at 0, 15, 30 and 60 min after injection.

### Indirect calorimetry

After a 24-hour adaption period, the respiratory quotient (RQ) of the animals was assessed using indirect calorimetry (Hartmann & Braun). The flow rate was 0.5 L/min, and the rates of oxygen consumption (VO_2_) and carbon dioxide production (VCO_2_) were measured at 22°C for 23 h. Water and food were freely available to the animals. The RQ is the quotient of VCO_2_/VO_2_. The following formulae were used to compute whole-body carbohydrate and fat oxidation rates (g/min): $carbohydrate\;oxidation\;rate=4.585\times \dot{V}CO_{2}(l/min)-3.226\times \dot{V}O_{2}(l/min)$ ; $fat\;oxidation\;rate=1.695\times \dot{V}O_{2}(l/min)-1.701\times \dot{V}CO_{2}(l/min)$ (24).

### ELISA quantification of sIL-6R in serum

To quantify mouse serum sIL-6R, the enzyme-linked immunosorbent assay (Mouse IL-6Ra DuoSet, cat. #DY1830, R&D Systems, Minneapolis, MN, USA) was used. Microtitre plates (Nunc maxi sorb, Sigma Aldrich, Munich, Germany) were incubated overnight with goat anti-mouse IL-6R capture antibody diluted in PBS (R&D Systems, 1.6 g/ml working concentration). After overnight incubation, the plates have been washed three times with 300 µL washing buffer (R&D Systems, cat. #WA12) prior blocking with 300 µL of PBS with 1% BSA for 1h at RT. Subsequently, diluted serum samples from sIL-6R^fl/fl^, sIL-6R^+/+^, IL-6R^-/-^ mice and wt littermates were added (100 µL/well) and incubate for 2h at RT. Plates were washed three times and biotinylated goat anti-mouse IL-6R mAbs (R&D Systems) were used to identify bound sIL-6R.

### Exercise

Mice aged 12 to 18 weeks ran on a treadmill 5 days a week for 6 weeks. The treadmill program was designed to gradually increase the intensity and duration of exercise, beginning with a warm-up and 10 m/min for 20 minutes totally (0° inclination) and progressing to 15-22 m/min for a total of 60 min of training with a 10° inclination.

### Statistics

Data are described as means ± SEMs. Significant differences were determined using one-way ANOVA, as indicated in the figure legends. P value <0.05 were considered statistically significant.

## Data availability statement

The raw data supporting the conclusions of this article will be made available by the authors, without undue reservation.

## Ethics statement

All experiments were approved by the Ethics Committee of the State Office for Nature, Environment and Consumer Protection NRW (LANUV, North Rhine-Westphalia, Germany – 84-02.04.2020.A278) and conducted at the animal facility of the German Diabetes Center.

## Author contributions

ARM. carried out the experiments and wrote the manuscript with support from JS. JS conceived the original idea and supervised the project. PR helped with mouse experiments and contributed to the project discussion. AC and HA-H helped in supervising the project. KB contributed to preparation of animal documentation. All authors provided constructive criticism and contributed to the development of the study. All authors contributed to the article and approved the submitted version.

## Funding

Funded by the Deutsche Forschungsgemeinschaft, Graduiertenkolleg VIVID.

## Acknowledgments

The authors thank RTG 2576 “Vivid- *In vivo* investigations towards the early development of type 2 diabetes” for funding this project. We thank Lena Espelage, Anna Scheel, Carina Heitmann and Birgit Knobloch for technical assistance.

## Conflict of interest

The authors declare that the research was conducted in the absence of any commercial or financial relationships that could be construed as a potential conflict of interest.

## Publisher’s note

All claims expressed in this article are solely those of the authors and do not necessarily represent those of their affiliated organizations, or those of the publisher, the editors and the reviewers. Any product that may be evaluated in this article, or claim that may be made by its manufacturer, is not guaranteed or endorsed by the publisher.
